# Molecular entities for the anti-melanoma effect of *Solanum nigrum*

**DOI:** 10.6026/973206300210679

**Published:** 2025-04-30

**Authors:** Neha Sakharkar, Ruchika Kaul-Ghanekar, Yingying Song, Jian Yang

**Affiliations:** 1 Bharati Vidyapeeth University, Pune - 411043, Maharashtra, India; 2 Symbiosis School of Biological Sciences (SSBS), Symbiosis Centre for Research and Innovation (SCRI), Symbiosis International (Deemed University), Pune - 412115, Maharashtra, India; 3 School of Chinese Medicine & School of Integrated Chinese and Western Medicine, Nanjing University of Chinese Medicine, Nanjing, 210023, China; 4 College of Pharmacy and Nutrition, University of Saskatchewan, Saskatoon, Saskatchewan S7N 5E5, Canada

**Keywords:** *Solanum nigrum*, melanoma, unique active ingredient, unique targets, gene expression

## Abstract

Prognosis for advanced and metastatic melanoma remains poor despite low incidence rate and early diagnosis. A significant number of melanoma patients use complementary and alternative medicines during their normal treatments in anticipating improving therapeutic efficacy. *Solanum nigrum* shows effective inhibitory activity against melanoma cells compared to *Hedyotis diffusa*, *Scutellaria barbata*, and *Lobelia chinensis*. Therefore, it is of interest to explore the anti-melanoma entities of *S. nigrum*. Our data show that three unique melanoma-related gene targets (CYP3A4, GBA2 and PTK6) for two unique active ingredients (diosgenin and solanocapsine) present in *S. nigrum* have potential role.

## Background:

Melanoma, which initiates from carcinogenesis of melanocytes, is the most serious type of skin cancer. Globally, 325,000 new melanoma cases and 57,000 melanoma-related deaths were estimated to occur in 2022 [1]. Although melanoma has a low incidence rate and is usually diagnosed in early stages, prognosis for metastatic melanoma is extremely poor [2, 3]. Currently, melanoma limited in the skin is usually removed by surgery. Melanoma spread beyond the skin is mainly treated with chemotherapy, radiotherapy, immunotherapy, and targeted therapy in addition to surgery [3]. To achieve better therapeutic efficacy, combination therapies, such as poly-chemotherapy and poly-immunotherapy, are also commonly administered in melanoma treatment [4]. Chemotherapy agents include dacarbazine, temozolomide, carboplatin, paclitaxel, cisplatin and vinblastine, whereas immunotherapy agents include immune checkpoint inhibitors such as ipilimumab, nivolumab and pembrolizumab and cytokines such as interferon α-2b and interleukin-2. However, these therapies are associated with high cost, severe toxicity, undesirable side effects, and rapid development of drug resistance (especially for metastatic melanoma) [5, 6, 7]. Thus, many melanoma patients, specifically patients from low-income countries, seek complementary and alternative medicine (CAM) treatments. A multicenter cross-sectional study of 1089 melanoma patients in Germany reported that about 40% of the patients showed an interest of using CAM and nearly 20% of the patients used CAM [8]. Natural health products and medicinal plant extracts have been widely used to treat different types of illnesses and diseases throughout the world. Various phytochemicals have also been shown to possess potent anticancer effect from both preclinical and clinical studies [9, 10-11]. Medicinal plants have emerged to be a valuable resource for identifying and developing anticancer therapeutics. In fact, 146 out of 174 anticancer drugs in the market between 1981 and 2014 have biological or natural product origins [12]. However, CAM usage (herbs, plant extracts, *etc.*) in cancer treatment faces several limitations. The major limitation is lack of understanding on the underlying molecular mechanisms of these products. The second limitation is insufficient molecular profiling of the plant components. Finally, information on molecular interactions among different components in CAM products and between CAM products and drugs, foods and regulator molecules in human body is very limited. Hence, it is of interest to explore CAM products, especially their respective mechanism of action (MOA), which will not only improve therapeutic efficacy of the treatments but also protect patients' safety. We evaluated the anticancer activities of *Hedyotis diffusa*, *Scutellaria barbata*, *Lobelia chinensis* and *Solanum nigrum* against human melanoma cell line A-375 in our previous study [13]. Surprisingly, only the water extract of *S. nigrum* (WESN) possessed potent activity and exhibited a synergistic effect with chemotherapy drug temozolomide, whereas the most-commonly used anticancer herbs, *H. diffusa* and *S. barbata*, elicited little activity. Therefore, it is of interest to explore the molecular entities for the anti-melanoma effect of *S. nigrum*.

## Methodology:

## Active ingredients and corresponding targets in human cells:

A methodology flowchart of the current study was presented in supplementary Figure 6 (see PDF). Information of bioactive compounds for each medicinal plant (Table 4) was retrieved from the TCMSP database and analysis platform (https://tcmspw.com/tcmsp.php) using *Hedyotis diffusa*, *Scutellaria barbata*, *Lobelia chinensis* or *Solanum nigrum* as a keyword. Oral bioavailability (OB ≥ 30%) and drug-likeness (DL ≥ 0.18) were set as the thresholds in identifying candidate active ingredients [14, 15]. For each candidate, we obtained the structural graphic (sdf format) from PubChem database (https://pubchem.ncbi.nlm.nih.gov/). Then, the sdf file was uploaded to the Swiss Target Prediction database (http://swisstargetprediction.ch/index.php) to identify potential interacting protein targets for the candidate compound [16]. Target protein names filtered with "Homo sapiens" and "Probability > 0" were downloaded. The protein targets for the active ingredients in each medicinal herb were summarized in supplementary Table 5.

## Melanoma-related targets:

Melanoma-related gene targets were retrieved from the GeneCards database (https://www.genecards.org/) using "melanoma" as a searching keyword. These targets were subsequently standardized in UniProt (https://www.uniprot.org/) with organism selected as "Homo sapiens" to obtain a list of human melanoma-related gene targets.

## Unique active ingredients:

Unique active ingredient refers to a bioactive compound that is present in one medicinal plant but not in the others. From supplementary Table 4, we identified 4, 23, 15 and 4 unique active ingredients in *H. diffusa*, *S. barbata*, *L. chinensis* and *S. nigrum*, respectively (Table 1).

## Unique targets for unique active ingredients:

For each medicinal plant, we first extracted the molecular targets for the unique active ingredients from supplementary Table 5. Then, unique molecular targets modulated by the unique active ingredients (Table 6) were obtained by removing shared molecular targets between/among medicinal plants. Finally, we identified unique melanoma-related targets for the unique active ingredients of each medicinal herb (Table 2) by extracting the common targets shared between the targets in supplementary Table 6 and human melanoma-related targets. We further carried out a reverse mapping of the 10 melanoma-related targets unique for *S. nigrum* for the corresponding unique active ingredients (Table 3).

## Gene expression profiling in melanoma:

From the above analyses, we identified 10 unique melanoma-related gene targets that were modulated by two unique active compounds, diosgenin and solanocapsine, in *S. nigrum*. We then analyzed the expression of these 10 genes in melanoma patients (TCGA-SKCM dataset) using online software GEPIA (http://gepia.cancer-pku.cn/) that can analyze RNA sequencing expression data from both TCGA and GTEx projects [17]. Three genes (CYP3A4, GBA2 and PTK6) were identified to be significantly downregulated in melanoma patients. We further calculated survival curves (Kaplan-Meier plots) for these three genes in melanoma patients (TCGA-SKCM). Only gene PTK6 exhibited survival benefits at low level of expression.

## Results:

*H. diffusa*, *S. barbata*, *L. chinensis* and *S. nigrum* are widely used to treat different types of cancer in traditional Chinese medicine (TCM). Particularly, *H. diffusa* and *S. barbata* are often used in pair, such as "kang ai ping wan" [18]. However, information on the anti-melanoma effect of these four medicinal herbs is very limited. Wang *et al.* reported that *S. nigrum* water extract could inhibit metastasis of melanoma cells in mice [19] and Chen *et al.* reported that the total flavonoids of *S. barbata* could inhibit melanoma growth by inducing autophagy and apoptosis [20]. However, Chen *et al.* used extracted flavonoids and it is unclear whether the content of *S. barbata* flavonoids in normal TCM formulas could reach to the level used in their experiment. Thus, we decided to undertake a comparative study of the four anticancer herbs against human melanoma A-375 cells and showed that only the water extract of *S. nigrum* (WESN) exhibited potent activity [13]. We further showed that WESN reduced intracellular ROS (reactive oxygen species) generation, which could lead to a cytostatic effect on the A-375 cells. Hence, in the current study, we embark on identifying unique melanoma targets for each herb and mapping them onto its respective active ingredients using a system pharmacology approach (Figure 6 - see PDF).

Traditional Chinese Medicine Systems Pharmacology (TCMSP) database and analysis platform is a database on systems pharmacology for herbal medicine, consisting of a collection of 499 Chinese herbs along with their respective bioactive ingredients, potential molecular targets and associated diseases [21]. Using this platform, we identified 7 active ingredients in *H. diffusa*, 29 active ingredients in *S. barbata*, 17 active ingredients in *L. chinensis* and 7 active ingredients in *S. nigrum* (Figure 1 - (see PDF) and Table 4). Only one compound, MOL000098 (quercetin), is shared among the four medicinal herbs. Unique active ingredients for each individual herb were summarized in Table 1. We identified 4 unique ingredients in *H. diffusa*, 23 in *S. barbata*, 15 in *L. chinensis* and 4 in *S. nigrum* (Table 1). Then, we extracted the molecular targets regulated by the active compounds of the four herbs (Figure 2 - (see PDF) and Table 5).

*H. diffusa*, *S. barbata*, *L. chinensis* and *S. nigrum* modulate 299, 299, 385 and 199 targets, respectively, out of which 146 targets are shared across the four herbs. We further identified that unique ingredients in *H. diffusa* modulate 82 unique targets, unique ingredients in *S. barbata* modulate 51 unique targets, unique ingredients in *L. chinensis* modulate 116 unique targets, and unique ingredients in *S. nigrum* modulate 27 unique targets, respectively (Table 6). To figure out why only *S. nigrum* is active against melanoma cells, we extracted 8144 melanoma-related gene targets from GeneCards and chose 4352 genes for further analysis after one cycle of median operation (score ≥ 0.69). Upon cross matching with targets modulated by the herbs, we identified 170 median melanoma-related targets from *H. diffusa*, 178 targets from *S. barbata*, 210 targets from *L. chinensis* and 112 targets from *S. nigrum*, respectively, out of which 87 gene targets are shared by all four herbs (Figure 3 - see PDF). Our further analysis showed that there are 41 gene targets unique for *H. diffusa*, 34 for *S. barbata*, 55 for *L. chinensis* and 10 for *S. nigrum* (Table 2).

Reverse mapping the 10 melanoma-related gene targets from *S. nigrum* to the active ingredients reveals that these 10 targets are modulated by 2 unique active compounds, diosgenin and solanocapsine (Table 1). This implicates that these two unique components from *S. nigrum* are likely to be responsible for the anticancer effect of WESN against the melanoma A-375 cells. Moreover, we performed a gene expression profiling for the 10 genes using TCGA-SKCM (skin cutaneous melanoma) dataset. Using | log_2_FC | ≥ 1 and p < 0.05 as the cutoff, three gene targets, CYP3A4, GBA2 and PTK6, were downregulated in skin cutaneous melanoma patients (Figure 4 - see PDF).

## Discussion:

As shown in Table 3, target genes CYP3A4 and PTK6 are modulated by diosgenin. CYP3A encodes cytochrome P450 3A4 (CYP3A4), one of the most important liver drug-metabolizing enzymes. CYP3A4 is also expressed in skin cells and involved in skin metabolism of drugs [22]. Our current analysis is consistent with a previous study by Sumantran *et al.* that gene CYP3A4 was downregulated in melanoma [23]. In addition, Manda *et al.* reported that diosgenin inhibited CYP3A4 at IC50 of 17 mM [24]. Thus, we may hypothesize that *S. nigrum* could further inhibit the already downregulated CYP3A4 in melanoma cells, which, in turn, would reduce skin metabolism of drugs including chemotherapy agents and be beneficial for melanoma treatments. However, *S. nigrum* contains many compounds and the function of diosgenin can be interfered by the other compounds. Further studies are needed to identify how *S. nigrum* modulates the expression and function (upregulation or downregulation) of the three identified genes in its TCM formulations. PTK6 encodes the non-receptor tyrosine kinase 6 (PTK6 or BRK). PTK6 is overexpressed in breast cancer and deemed as an oncogene to promote cancer cell growth, survival, and migration [25- 26]. However, its expression is downregulated in melanoma patients. Recently, Fei and Chen proposed that PTK6, CAPNS1, DAPK2 and PARP1 formed an autophagy-related risk model, which could be used to evaluate prognosis of melanoma patients [27]. Furthermore, Kaplan Meier survival plot for patients included in the TCGA-SKCM dataset shows that high expression of PTK6 is associated with poor prognosis (Figure 5 - see PDF). Hence, we suggest that the downregulation of PTK6 in SKCM patients is potentially a response for maintaining homeostasis as autophagy has been reported to play an important role in maintaining skin homeostasis at cellular level [28, 29, 30-31]. Other than CYP3A4 and PTK6, other genes such as AKT1 and CDK1 have been identified as targets for diosgenin from both our current analysis and previous reports [32, 33]. However, these targets are unlikely to be responsible for the anti-melanoma activity of *S. nigrum* as they are also regulated by other medicinal plants such as *H. diffusa*, *S. barbata* and *L. chinensis*. Therefore, we conclude that genes CYP3A4 and PTK6, which are modulated by diosgenin, are likely to be the major targets conferring *S. nigrum* the anti-melanoma effect.

The third downregulated gene target GBA2 encodes non-lysosomyl glucosylceramidase β2 (GBA2), which catalyzes the hydrolysis of glucosylceramide into ceramide and glucose. It is modulated by solanocapsine, which is another unique active ingredient in *S. nigrum*. It has been shown that inducing GBA2 expression would promote glucosylceramide hydrolysis and impair melanoma growth in a mouse xenograft model [34]. Ceramide is an essential component of the highly evolutionarily conserved sphingolipid metabolism pathway. It forms a rheostat with sphingosine-1-phosphate (S1P), another key and potent sphingolipid metabolite [35]. The ceramide-S1P rheostat regulate a broad range of biological functions, such as ROS generation, cell proliferation and survival, cell apoptosis and cell autophagy [36, 37-38]. A shift of the rheostat towards ceramide side induces cell apoptosis and autophagy. Thus, GBA2 is highly like to exert its functions on cancer cell growth via regulating ceramide level in the ceramide-S1P rheostat. However, the modulation mechanism by solanocapsine on either GBA2 or cancer cell growth is still unknown. Further studies are warranted to identify the biological pathways regulated solanocapsine in human body.

## Conclusion:

The anti-melanoma effect of *S. nigrum* is likely through the modulation of three unique melanoma-related gene targets (CYP3A4, GBA2 and PTK6) by its two unique active ingredients (diosgenin and solanocapsine). However, it should be noted that there are two major limitations: 1) the anti-melanoma effect of *S. nigrum* is possibly via the coordination of multiple active ingredients and may not be accurately represented by modulations from a single active ingredient and 2) crosstalk between different signaling pathways was not studied in the current research. Therefore, future transcriptomic and/or proteomic studies may provide a more accurate mechanism of action (MOA) for *S. nigrum* and illustrate the different types of crosstalk among signaling pathways regulated by *S. nigrum*.

## Competing interests:

The authors declared no competing interests with respect to the research, authorship, and/or publication of this article.

## Funding:

The authors disclosed no financial support for the research, authorship, and/or publication of this article.

## Ethical approval:

Not applicable.

## Author contributions:

Research idea: J Yang; Extraction of information on herbal active ingredients: Y Song; Analysis of melanoma targets: N Sakharkar; Data analysis: N Sakharkar, R Kaul-Ghanekar, Y Song, J Yang; Manuscript writing: N Sakharkar, J Yang.

## Figures and Tables

**Table 1:** Unique active ingredients identified in *Hedyotis diffusa*, Scutellaria barbata, *Lobelia chinensis* and Solanum nigrum

***H. diffusa***	***S. barbata***	***L. chinensis***	***S. nigrum***
**4**	**23**	**15**	**4**
MOL001646	MOL001040	MOL011678	MOL002058
MOL001659	MOL012245	MOL001790	MOL002773
MOL001663	MOL012246	MOL012207	MOL000546
MOL001670	MOL012248	MOL012208	MOL007356
	MOL012250	MOL012209	
	MOL012251	MOL012216	
	MOL012252	MOL012225	
	MOL002776	MOL009653	
	MOL012254	MOL001689	
	MOL012266	MOL002341	
	MOL001973	MOL002881	
	MOL012269	MOL003044	
	MOL012270	MOL000422	
	MOL000173	MOL005530	
	MOL001735	MOL009009	
	MOL001755		
	MOL002714		
	MOL002719		
	MOL002915		
	MOL000351		
	MOL005190		
	MOL005869		
	MOL008206		

**Table 2:** Unique human melanoma-related gene targets regulated by the unique active ingredients in *Hedyotis diffusa*, *Scutellaria barbata*, *Lobelia chinensis* and *Solanum nigrum*

***H. diffusa***	***S. barbata***	***L. chinensis***	***S. nigrum***
**41**	**34**	**55**	**10**
ACP1	ADAM17	ACKR3	AGL
ATM	ATP12A	ADAM33	AKT2
CCND2	BCL2L1	ADRB3	CYP24A1
CCND3	BRAF	AKT3	CYP3A4
CD81	CNOT7	AURKA	FASN
CDC25B	CTSD	BRD4	GBA2
CDK8	CTSL	CCKAR	PTK6
CMA1	DYRK1B	CCKBR	RASGRP3
CXCR3	EIF2AK2	CCR3	SMO
FABP5	ERCC5	CCR4	UGCG
FAP	ERN1	CDC25C	
FTO	FEN1	CHRM3	
GABRA5	FLT1	CHRM5	
GAPDH	FLT4	CREBBP	
GSTP1	HSP90AB1	CXCR2	
HDAC6	HSP90B1	CYP2D6	
ICAM1	IGFBP3	DPP4	
IDH1	IKBKB	DRD2	
KAT2B	KCNMA1	EGLN1	
KCNN2	KDM1A	EIF2AK3	
KCNN3	MMP14	ELAVL1	
KDM5B	MMP16	EZH2	
LIMK1	NQO1	FYN	
MAP2K1	NTRK2	HDAC1	
NCSTN	PDE4A	HDAC8	
PDE4D	PGF	HRH1	
PDPK1	PRKCB	HRH2	
PLEC	PRKCE	IKBKE	
PSEN1	RET	IMPDH2	
PTGER4	SNCA	JAK1	
PTK2B	TDP1	JAK2	
PTP4A3	TRPV1	JAK3	
SELE	TUBB3	MAP4K4	
SIRT2	VEGFA	MAPK1	
STAT3		MAPK11	
TGFBR1		MME	
TGM2		MPI	
TLR9		MTAP	
TNFRSF1A		MTOR	
UBA2		NTRK1	
VCAM1		PAK4	
		PLD1	
		PRKCA	
		PRMT1	
		ROCK2	
		RPS6KB1	
		SCARB1	
		SPHK1	
		SSTR5	
		TBK1	
		TK1	
		TP53	
		TRPV3	
		TTK	
		YES1	

**Table 3:** Corresponding unique active ingredients that regulate the 10 unique melanoma-related gene targets for *Solanum nigrum*.

**Unique melanoma-related gene targets**	**Respective active ingredients**	
AGL	MOL007356	solanocapsine
AKT2	MOL007356	solanocapsine
CYP24A1	MOL000546	diosgenin
CYP3A4	MOL000546	diosgenin
FASN	MOL000546	diosgenin
GBA2	MOL007356	solanocapsine
PTK6	MOL000546	diosgenin
RASGRP3	MOL000546	diosgenin
SMO	MOL000546	diosgenin
UGCG	MOL007356	solanocapsine

**Table 4:** Major active ingredients identified in *Hedyotis diffusa*, *Scutellaria barbata*, *Lobelia chinensis* and *Solanum nigrum*

***Hedyotis diffusa***	***Scutellaria barbata***	***Lobelia chinensis***	***Solanum nigrum***
**7**	**29**	**17**	**7**
MOL001646	MOL001040	MOL011678	MOL002058
MOL001659	MOL012245	MOL001790	MOL002773
MOL001663	MOL012246	MOL012207	MOL000359
MOL001670	MOL012248	MOL012208	MOL000546
MOL000449	MOL012250	MOL012209	MOL007356
MOL000358	MOL012251	MOL012216	MOL000953
MOL000098	MOL012252	MOL012225	MOL000098
	MOL002776	MOL009653	
	MOL012254	MOL001689	
	MOL000953	MOL002341	
	MOL000358	MOL002881	
	MOL012266	MOL003044	
	MOL001973	MOL000422	
	MOL012269	MOL005530	
	MOL012270	MOL000006	
	MOL000449	MOL009009	
	MOL000173	MOL000098	
	MOL001735		
	MOL001755		
	MOL002714		
	MOL002719		
	MOL002915		
	MOL000351		
	MOL000359		
	MOL005190		
	MOL005869		
	MOL000006		
	MOL008206		
	MOL000098		

**Table 5:** Molecular targets (human) modulated by major active ingredients of *Hedyotis diffusa*, *Scutellaria barbata*, *Lobelia chinensis* and *Solanum nigrum*

***Hedyotis diffusa***	***Scutellaria barbata***	***Lobelia chinensis***	***Solanum nigrum***
**299**	**299**	**385**	**199**
ABCB1	ABCB1	ABCB1	ABCC1
ABCC1	ABCC1	ABCC1	ABCG2
ABCG2	ABCG2	ABCG2	ACHE
ABL1	ACHE	ABL1	ADORA1
ACHE	ADAM17	ACHE	ADORA2A
ACP1	ADCY5	ACKR3	AGL
ADAMTS5	ADORA1	ADAM33	AHR
ADORA1	ADORA2A	ADORA1	AKR1A1
ADORA2A	ADORA3	ADORA2A	AKR1B1
ADORA3	ADRA2A	ADORA3	AKR1B10
ADRA1A	ADRA2C	ADRA1A	AKR1C1
ADRA1B	AHR	ADRA1B	AKR1C2
ADRA1D	AKR1A1	ADRA1D	AKR1C3
AHR	AKR1B1	ADRA2A	AKR1C4
AKR1A1	AKR1B10	ADRA2B	AKT1
AKR1B1	AKR1C1	ADRA2C	AKT2
AKR1B10	AKR1C2	ADRB3	ALK
AKR1C1	AKR1C3	AHR	ALOX12
AKR1C2	AKR1C4	AKR1A1	ALOX15
AKR1C3	AKT1	AKR1B1	ALOX5
AKR1C4	ALDH2	AKR1B10	APEX1
AKT1	ALK	AKR1C1	APP
ALK	ALOX12	AKR1C2	AR
ALOX12	ALOX15	AKR1C3	ARG1
ALOX15	ALOX5	AKR1C4	AURKB
ALOX5	AMY1A	AKT1	AVPR1A
ALOX5AP	APEX1	AKT3	AVPR2
APEX1	APP	ALDH2	AXL
APH1A	AR	ALK	BACE1
APH1B	ARG1	ALOX12	BCHE
APP	ATP12A	ALOX15	CA1
AR	AURKB	ALOX5	CA12
ARG1	AVPR2	AMY1A	CA13
ATM	AXL	APEX1	CA14
AURKB	BACE1	APP	CA2
AVPR2	BCHE	AR	CA3
AXL	BCL2	ARG1	CA4
BACE1	BCL2L1	AURKA	CA5A
BCHE	BMP1	AURKB	CA6
BCL2	BRAF	AVPR2	CA7
CA1	CA1	AXL	CA9
CA12	CA12	BACE1	CAMK2B
CA13	CA13	BCHE	CCNB1
CA14	CA14	BCL2	CCNB2
CA2	CA2	BMP1	CCNB3
CA3	CA3	BRD4	CD38
CA4	CA4	CA1	CDK1
CA5A	CA5A	CA12	CDK2
CA6	CA5B	CA13	CDK5
CA7	CA6	CA14	CDK5R1
CA9	CA7	CA2	CDK6
CACNA1B	CA9	CA3	CES2
CAMK2B	CALM1	CA4	CFD
CAPN1	CAMK2B	CA5A	CHRM2
CCNB1	CBR1	CA5B	CNR2
CCNB2	CCNA1	CA6	COL4A3BP
CCNB3	CCNA2	CA7	CPT1A
CCNC	CCNB1	CA9	CPT1B
CCND1	CCNB2	CACNA1H	CPT2
CCND2	CCNB3	CALM1	CSNK2A1
CCND3	CCR1	CAMK2B	CXCR1
CCNE1	CCR5	CBR1	CYP17A1
CCNE2	CD38	CCKAR	CYP19A1
CCNT1	CDC25A	CCKBR	CYP1B1
CCR5	CDK1	CCNA1	CYP24A1
CD38	CDK2	CCNA2	CYP2C19
CD81	CDK5	CCNB1	CYP2C9
CDC25A	CDK5R1	CCNB2	CYP3A4
CDC25B	CDK6	CCNB3	CYP51A1
CDK1	CES1	CCND1	DAPK1
CDK2	CES2	CCNE1	DGAT1
CDK4	CFTR	CCNE2	DHCR7
CDK5	CHEK1	CCR1	DRD4
CDK5R1	CHRM2	CCR3	EGFR
CDK6	CHRNA7	CCR4	ESR1
CDK8	CISD1	CCR5	ESR2
CDK9	CLK1	CD38	ESRRA
CES2	CNOT7	CDC25A	F2
CHEK1	CSNK2A1	CDC25C	FASN
CHRM1	CTSB	CDK1	FDFT1
CHRM2	CTSD	CDK2	FLT3
CMA1	CTSK	CDK4	G6PD
CNR1	CTSL	CDK5	GAA
CSF1R	CXCR1	CDK5R1	GBA
CSNK2A1	CYP17A1	CDK6	GBA2
CTSB	CYP19A1	CDK9	GLO1
CTSS	CYP1A1	CES1	GLRA1
CTSV	CYP1A2	CES2	GPR35
CXCR1	CYP1B1	CFTR	GSK3B
CXCR3	CYP2C19	CHEK1	HMGCR
CYP11B1	CYP51A1	CHRM1	HSD11B1
CYP11B2	DAPK1	CHRM2	HSD11B2
CYP17A1	DHCR7	CHRM3	HSD17B1
CYP19A1	DNM1	CHRM4	HSD17B2
CYP1A1	DNMT1	CHRM5	IGF1R
CYP1A2	DRD4	CHRNA3	IL2
CYP1B1	DUSP3	CHRNA4	INSR
CYP2C19	DYRK1A	CHRNA6	KCNA3
CYP51A1	DYRK1B	CHRNA7	KDM4E
DAPK1	EDNRA	CHRNB2	KDR
DHCR7	EGFR	CHRNB3	KIT
DRD4	EIF2AK2	CHRNB4	MAOA
DUSP3	ELANE	CNR2	MAPK14
EGFR	EPHB4	CREBBP	MAPK8
ELANE	ERCC5	CSF1R	MAPK9
EPHB4	ERN1	CSNK2A1	MAPT
ESR1	ESR1	CTSK	MCL1
ESR2	ESR2	CXCR1	MDM2
ESRRA	ESRRA	CXCR2	MET
F2	F2	CYP17A1	MGAM
FAAH	FAAH	CYP19A1	MMP12
FABP1	FABP1	CYP1A1	MMP13
FABP3	FDFT1	CYP1A2	MMP2
FABP4	FEN1	CYP1B1	MMP3
FABP5	FFAR1	CYP2C19	MMP9
FAP	FGFR1	CYP2D6	MPG
FDFT1	FLT1	CYP2J2	MPO
FFAR1	FLT3	CYP51A1	MTNR1A
FGFR1	FLT4	DAPK1	MTNR1B
FLT3	FNTA	DHCR7	MYLK
FNTA	FNTB	DNM1	NEK2
FNTB	FUT4	DNMT1	NEK6
FTO	FUT7	DPP4	NOS2
G6PD	G6PD	DRD1	NOX4
GABRA1	GCKR	DRD2	NPC1L1
GABRA2	GLO1	DRD3	NR1H2
GABRA3	GLRA1	DRD4	NR1H3
GABRA5	GPR35	DRD5	NR1H4
GABRB3	GRK2	DUSP3	NR1I3
GABRG2	GRK6	DUT	NR3C1
GAPDH	GRM2	DYRK1A	NUAK1
GLO1	GRM5	EBP	OPRL1
GLRA1	GSK3B	EDNRA	OPRM1
GPBAR1	GUSB	EGFR	PABPC1
GPR35	HIF1A	EGLN1	PARP1
GRIK1	HMGCR	EIF2AK3	PCSK7
GRIK2	HNF4A	ELAVL1	PDE10A
GRM4	HSD11B1	ESR1	PDGFRB
GRM5	HSD11B2	ESR2	PGK1
GSK3B	HSD17B1	ESRRA	PIK3CA
GSTP1	HSD17B2	EZH2	PIK3CB
HCK	HSD17B3	F2	PIK3CD
HCRTR1	HSP90AA1	F5	PIK3CG
HCRTR2	HSP90AB1	FBP1	PIK3R1
HDAC6	HSP90B1	FCER2	PIM1
HMGCR	IDO1	FDFT1	PKN1
HSD11B1	IGF1R	FFAR1	PLA2G1B
HSD11B2	IGFBP3	FGFR1	PLA2G2C
HSD17B1	IKBKB	FLT3	PLK1
HSD17B2	IL2	FUCA1	POLB
HSP90AA1	INSR	FUT4	PPARA
HTR1A	KCNH2	FUT7	PPARD
HTR1B	KCNMA1	FYN	PPARG
HTR2A	KDM1A	G6PD	PREP
HTR2B	KDM4E	GBA	PTAFR
ICAM1	KDM5A	GLO1	PTGER1
IDH1	KDR	GLRA1	PTGER2
IDO1	KIT	GPR142	PTGES
IGF1R	KLK1	GPR35	PTGS1
INSR	KLK2	GRIK1	PTK2
KAT2B	LCK	GRIN1	PTK6
KCNN1	MAOA	GRIN2B	PTPN1
KCNN2	MAOB	GRK6	PTPN2
KCNN3	MAPK14	GRM2	PTPN6
KDM4E	MAPK3	GRM5	PTPRS
KDM5A	MAPT	GSK3B	PYGL
KDM5B	MCL1	GUSB	RASGRP3
KDR	MET	H1F0	RBP4
LCK	MMP1	HCK	RORA
LIMK1	MMP12	HCN1	RORC
LRRK2	MMP13	HCN4	SERPINA6
LTB4R	MMP14	HDAC1	SHBG
MAOA	MMP16	HDAC8	SHH
MAP2K1	MMP2	HIF1A	SI
MAPK8	MMP3	HMGCR	SIGMAR1
MAPT	MMP7	HRH1	SLC22A12
MCL1	MMP8	HRH2	SLC22A2
MDM2	MMP9	HRH3	SLC47A1
MET	MPG	HSD11B1	SLC5A2
MGLL	MPO	HSD11B2	SLC6A2
MMP1	MYLK	HSD17B1	SLC6A4
MMP12	NAE1	HSD17B2	SMO
MMP13	NEK2	HSP90AA1	SQLE
MMP2	NEK6	HTR1A	SRC
MMP3	NMUR2	HTR1B	SREBF2
MMP9	NOS2	HTR1F	SYK
MPG	NOX4	HTR2A	TACR1
MPO	NPC1L1	HTR2B	TBXAS1
MYLK	NQO1	HTR2C	TERT
NAAA	NQO2	HTR4	TNKS
NCSTN	NR1H2	HTR5A	TNKS2
NEK2	NR1H3	HTR6	TOP1
NEK6	NR1I2	HTR7	TOP2A
NOS2	NR1I3	IGF1R	TTR
NOX4	NR3C1	IKBKE	TYR
NPC1L1	NR3C2	IL2	UGCG
NR1H2	NTRK2	IMPDH2	UGT2B7
NR1H3	NUAK1	INSR	VDR
NR1H4	ODC1	JAK1	XDH
NR1I3	OPRD1	JAK2	
NR3C1	OPRM1	JAK3	
NR3C2	PARP1	KCNA3	
NUAK1	PDE4A	KCNH2	
P2RX7	PDE4C	KCNJ1	
PABPC1	PDE5A	KCNK2	
PARP1	PDGFRA	KCNK3	
PDE10A	PDGFRB	KCNQ1	
PDE1B	PFKFB3	KDM4E	
PDE2A	PGD	KDR	
PDE4B	PGF	KIT	
PDE4D	PGR	KLK1	
PDE5A	PIK3CG	KLK2	
PDE9A	PIK3R1	LCK	
PDGFRA	PIM1	LRRK2	
PDGFRB	PIM2	MAOA	
PDPK1	PIM3	MAOB	
PFKFB3	PKN1	MAP3K12	
PGGT1B	PLA2G10	MAP4K4	
PGR	PLA2G1B	MAPK1	
PIK3CA	PLA2G2A	MAPK11	
PIK3CD	PLA2G5	MAPK14	
PIK3CG	PLA2G7	MAPK3	
PIK3R1	PLG	MAPK8	
PIM1	PLK1	MAPK9	
PIM2	POLA1	MAPKAPK2	
PKN1	POLB	MAPT	
PLA2G1B	PPARA	MCL1	
PLEC	PPARD	MET	
PLK1	PPARG	MKNK1	
POLB	PREP	MKNK2	
PPARA	PRKCB	MME	
PPARD	PRKCD	MMP1	
PPARG	PRKCE	MMP12	
PPOX	PRKCG	MMP13	
PREP	PRKCH	MMP2	
PRKCH	PRKCQ	MMP3	
PRKDC	PTGER1	MMP7	
PSEN1	PTGER2	MMP8	
PSEN2	PTGER3	MMP9	
PSENEN	PTGES	MPG	
PTGER1	PTGS1	MPI	
PTGER2	PTGS2	MPO	
PTGER4	PTK2	MTAP	
PTGES	PTPN1	MTNR1A	
PTGIR	PTPN11	MTNR1B	
PTGS1	PTPN2	MTOR	
PTGS2	PTPN6	MYLK	
PTK2	PTPRS	NAE1	
PTK2B	PYGL	NEK1	
PTP4A3	RET	NEK2	
PTPN1	RORA	NEK6	
PTPN11	RORC	NMUR2	
PTPN2	RPS6KA3	NOS2	
PTPN6	RPS6KA5	NOX4	
PTPRF	RXRA	NPBWR1	
PTPRS	SERPINA6	NPC1L1	
PYGL	SERPINE1	NQO2	
PYGM	SHBG	NR1H2	
RORA	SHH	NR1H3	
RORC	SIGMAR1	NR1I3	
SAE1	SLC22A12	NR3C2	
SCD	SLC5A2	NTRK1	
SCN4A	SLC6A2	NUAK1	
SELE	SLC6A3	ODC1	
SERPINA6	SLC6A4	OPRD1	
SHBG	SNCA	OPRK1	
SIGMAR1	SQLE	OPRL1	
SIRT2	SRC	OPRM1	
SLC22A12	SRD5A1	P2RX3	
SLC6A2	SRD5A2	P2RX7	
SLC6A3	SREBF2	PAK4	
SLC6A4	ST3GAL3	PARP1	
SOAT1	ST6GAL1	PARP3	
SQLE	STAT1	PDE10A	
SRC	STS	PDE1C	
SRD5A2	SYK	PDE2A	
SREBF2	TACR2	PDE7A	
STAT3	TAS2R31	PDE9A	
SYK	TBXA2R	PDGFRB	
TBXAS1	TBXAS1	PFKFB3	
TERT	TDP1	PGD	
TGFBR1	TERT	PI4KB	
TGM2	TNF	PIK3CA	
THRA	TNKS	PIK3CB	
THRB	TNKS2	PIK3CD	
TLR9	TOP1	PIK3CG	
TNFRSF1A	TOP2A	PIK3R1	
TNKS	TRPA1	PIM1	
TNKS2	TRPV1	PIM2	
TOP1	TTL	PIM3	
TOP2A	TTR	PKN1	
TRPA1	TUBB1	PKN2	
TTR	TUBB3	PLA2G10	
TYR	TYR	PLA2G1B	
UBA2	UGT2B7	PLA2G2A	
UGT2B7	VDR	PLA2G5	
VCAM1	VEGFA	PLA2G7	
VDR	WEE1	PLD1	
XDH	XDH	PLD2	
		PLG	
		PLK1	
		POLB	
		POLR1A	
		PPARA	
		PPARD	
		PPARG	
		PRKCA	
		PRKCD	
		PRKCQ	
		PRKDC	
		PRMT1	
		PTAFR	
		PTGER1	
		PTGER2	
		PTGES	
		PTGS1	
		PTGS2	
		PTK2	
		PTPN1	
		PTPN2	
		PTPN6	
		PTPRS	
		PYGL	
		REN	
		ROCK2	
		RORA	
		RORC	
		RPS6KA3	
		RPS6KB1	
		RXRA	
		SCARB1	
		SCN1A	
		SCN9A	
		SERPINA6	
		SERPINE1	
		SGPL1	
		SHBG	
		SIGMAR1	
		SLC18A2	
		SLC18A3	
		SLC22A12	
		SLC5A2	
		SLC6A1	
		SLC6A2	
		SLC6A3	
		SLC6A4	
		SOAT1	
		SOAT2	
		SPHK1	
		SPHK2	
		SQLE	
		SRC	
		SRD5A1	
		SRD5A2	
		SREBF2	
		SSTR5	
		ST3GAL3	
		ST6GAL1	
		STAT1	
		SYK	
		TAAR1	
		TACR1	
		TACR2	
		TAS2R31	
		TBK1	
		TERT	
		THRA	
		THRB	
		TK1	
		TNF	
		TNKS	
		TNKS2	
		TOP1	
		TOP2A	
		TP53	
		TRPA1	
		TRPV3	
		TTK	
		TTR	
		TYR	
		UGT2B7	
		VDR	
		WEE1	
		XDH	
		YES1	

**Table 6:** Unique molecular targets (human) modulated by the unique active ingredients of *Hedyotis diffusa*, *Scutellaria barbata*, *Lobelia chinensis* and *Solanum nigrum*

***Hedyotis diffusa***	***Scutellaria barbata***	***Lobelia chinensis***	***Solanum nigrum***
**82**	**51**	**116**	**27**
ACP1	ADAM17	ACKR3	AGL
ADAMTS5	ADCY5	ADAM33	AKT2
ALOX5AP	ATP12A	ADRA2B	AVPR1A
APH1A	BCL2L1	ADRB3	CFD
APH1B	BRAF	AKT3	COL4A3BP
ATM	CISD1	AURKA	CPT1A
CACNA1B	CLK1	BRD4	CPT1B
CAPN1	CNOT7	CACNA1H	CPT2
CCNC	CTSD	CCKAR	CYP24A1
CCND2	CTSL	CCKBR	CYP2C9
CCND3	DYRK1B	CCR3	CYP3A4
CCNT1	EIF2AK2	CCR4	DGAT1
CD81	ERCC5	CDC25C	FASN
CDC25B	ERN1	CHRM3	GAA
CDK8	FEN1	CHRM4	GBA2
CMA1	FLT1	CHRM5	MGAM
CNR1	FLT4	CHRNA3	PCSK7
CTSS	GCKR	CHRNA4	PGK1
CTSV	GRK2	CHRNA6	PLA2G2C
CXCR3	HNF4A	CHRNB2	PTK6
CYP11B1	HSD17B3	CHRNB3	RASGRP3
CYP11B2	HSP90AB1	CHRNB4	RBP4
FABP3	HSP90B1	CREBBP	SI
FABP4	IGFBP3	CXCR2	SLC22A2
FABP5	IKBKB	CYP2D6	SLC47A1
FAP	KCNMA1	CYP2J2	SMO
FTO	KDM1A	DPP4	UGCG
GABRA1	MMP14	DRD1	
GABRA2	MMP16	DRD2	
GABRA3	NQO1	DRD3	
GABRA5	NR1I2	DRD5	
GABRB3	NTRK2	DUT	
GABRG2	PDE4A	EBP	
GAPDH	PDE4C	EGLN1	
GPBAR1	PGF	EIF2AK3	
GRIK2	POLA1	ELAVL1	
GRM4	PRKCB	EZH2	
GSTP1	PRKCE	F5	
HCRTR1	PRKCG	FBP1	
HCRTR2	PTGER3	FCER2	
HDAC6	RET	FUCA1	
ICAM1	RPS6KA5	FYN	
IDH1	SNCA	GPR142	
KAT2B	STS	GRIN1	
KCNN1	TBXA2R	GRIN2B	
KCNN2	TDP1	H1F0	
KCNN3	TRPV1	HCN1	
KDM5B	TTL	HCN4	
LIMK1	TUBB1	HDAC1	
LTB4R	TUBB3	HDAC8	
MAP2K1	VEGFA	HRH1	
MGLL		HRH2	
NAAA		HRH3	
NCSTN		HTR1F	
PDE1B		HTR2C	
PDE4B		HTR4	
PDE4D		HTR5A	
PDPK1		HTR6	
PGGT1B		HTR7	
PLEC		IKBKE	
PPOX		IMPDH2	
PSEN1		JAK1	
PSEN2		JAK2	
PSENEN		JAK3	
PTGER4		KCNJ1	
PTGIR		KCNK2	
PTK2B		KCNK3	
PTP4A3		KCNQ1	
PTPRF		MAP3K12	
PYGM		MAP4K4	
SAE1		MAPK1	
SCD		MAPK11	
SCN4A		MAPKAPK2	
SELE		MKNK1	
SIRT2		MKNK2	
STAT3		MME	
TGFBR1		MPI	
TGM2		MTAP	
TLR9		MTOR	
TNFRSF1A		NEK1	
UBA2		NPBWR1	
VCAM1		NTRK1	
		OPRK1	
		P2RX3	
		PAK4	
		PARP3	
		PDE1C	
		PDE7A	
		PI4KB	
		PKN2	
		PLD1	
		PLD2	
		POLR1A	
		PRKCA	
		PRMT1	
		REN	
		ROCK2	
		RPS6KB1	
		SCARB1	
		SCN1A	
		SCN9A	
		SGPL1	
		SLC18A2	
		SLC18A3	
		SLC6A1	
		SOAT2	
		SPHK1	
		SPHK2	
		SSTR5	
		TAAR1	
		TBK1	
		TK1	
		TP53	
		TRPV3	
		TTK	
		YES1	
